# Prospective, multicenter study of antimicrobial-coated, noncrosslinked, acellular porcine dermal matrix (XenMatrix™ AB Surgical Graft) for hernia repair in all centers for disease control and prevention wound classes: 24-month follow-up cohort

**DOI:** 10.1097/MS9.0000000000000695

**Published:** 2023-04-19

**Authors:** Obeid N. IIahi, George Velmahos, Jeffrey E. Janis, Stephen J. Kovach, Susan F. McLean, Reza Askari, Courtney A. Sommer, Suresh Agarwal, Jahnavi Srinivasan, Alex K. Wong, Andrea Pakula, Edward P. Miranda, Kerry Breen, Mark Shapiro, Corey R. Deeken, R. Cody Stringer, J. Reed McGraw, Corey Bascone, Robert G. Martindale

**Affiliations:** a Washington University School of Medicine; b Covalent Bio, LLC, St. Louis, MO; c Massachusetts General Hospital; d Brigham and Women’s Hospital, Boston, MA; e The Ohio State University, Columbus, OH; f University of Pennsylvania, Philadelphia, PA; g Texas Tech University Health Science Center School of Medicine, El Paso, TX; h Duke University Medical Center, Durham, NC; i Emory University, Atlanta, GA; j City of Hope National Medical Center, Duarte, CA; k Kern Medical, Bakersfield; l University of California San Francisco, San Francisco, CA; m Oregon Health & Science University, Portland, OR

**Keywords:** hernia repair, minocycline, porcine dermal matrix, rifampin, surgical site occurrence, XenMatrix AB surgical graft

## Abstract

Prospective, multicenter, single-arm study of antimicrobial-coated, noncrosslinked, acellular porcine dermal matrix (AC-PDM) in a cohort involving all centers for disease control and prevention wound classes in ventral/incisional midline hernia repair (VIHR).

**Materials and methods:**

Seventy-five patients (mean age 58.6±12.7 years; BMI 31.3±4.9 kg/m^2^) underwent ventral/incisional midline hernia repair with AC-PDM. Surgical site occurrence (SSO) was assessed in the first 45 days post-implantation. Length of stay, return to work, hernia recurrence, reoperation, quality of life, and SSO were assessed at 1, 3, 6, 12, 18, and 24 months.

**Results:**

14.7% of patients experienced SSO requiring intervention within 45 days post-implantation, and 20.0% thereafter (>45 d post-implantation). Recurrence (5.8%), definitely device-related adverse events (4.0%), and reoperation (10.7%) were low at 24 months; all quality-of-life indicators were significantly improved compared to baseline.

**Conclusion:**

AC-PDM exhibited favourable results, including infrequent hernia recurrence and definitely device-related adverse events, with reoperation and SSO comparable to other studies, and significantly improved quality of life.

## Introduction

Highlights

- Ventral or incisional midline hernia repair in all centers for disease control and prevention wound classes.
- Antimicrobial-coated, noncrosslinked acellular porcine dermal matrix.
- Outcomes: surgical site occurrence, recurrence, reoperation, quality of life, length of stay, return to work.
- Low hernia recurrence (5.8%) and definitely device-related adverse events (4.0%).
- Expected reoperation (10.7%) and surgical site occurrence (K–M: 14.7% ≤45 days and 20.0% >45 days).

Over the past several decades, there have been numerous advancements in the field of ventral hernia repair in the areas of surgical techniques and biomaterials utilized for abdominal wall reconstruction^1^. Initially, materials such as silver and tantalum were utilized for hernia repair, followed by permanent synthetic polymers such as polypropylene, polyester, and polytetrafluoroethylene^2^–^4^. In more recent years, biological tissue-derived extracellular matrix scaffolds^5^ were popularized, along with resorbable polymer matrices^6^,^7^ and “hybrid” combinations of extracellular matrix scaffolds paired with permanent or resorbable components^8^. Many hernia repair materials are available in an uncoated “bare” configuration, but various anti-adhesive barriers/coatings have also been developed to minimize tissue attachment to the underlying scaffold^1^. Most recently, antibacterial-coated materials have been explored, including both synthetic^9^ and biological tissue-derived scaffolds^10^ in a variety of *in vitro* 11,12 and preclinical models^13^,^14^. Interestingly, a protective effect of the coating was demonstrated when an antimicrobial-coated, noncrosslinked, porcine acellular dermal matrix material (AC-PDM, XenMatrix™ AB Surgical Graft, C. R. Bard / Davol, Inc.) was utilized in several preclinical models involving contaminated wound environments^13^,^14^. In the first, a rabbit bacterial inoculation model, AC-PDM demonstrated antimicrobial efficacy against clinically-isolated MRSA and *E. coli* compared with uncoated materials^14^. Zero adherent colony forming units were observed at seven days post-implantation/inoculation for both inoculates^14^. Significantly lower inflammation scores and neutrophil scores at 7 days post-implantation/inoculation were also observed, as well as reduced abscess formation and bacterial-induced neutrophil response^14^. In another rabbit bacterial inoculation model^13^, AC-PDM also completely inhibited abscess formation and bacterial colonization and significantly reduced inflammation in MRSA and *E. coli* inoculated animals at 2-week and 8-week post-implantation^13^.

Despite these promising results, it remains unknown how these preclinical data may translate into clinical outcomes. Thus, the current study was designed to determine the properties of an AC-PDM in a cohort of patients up to 24 months post-implantation. The objective of the study was to prospectively evaluate the clinical performance of an AC-PDM in a multicenter clinical trial. Unlike other studies involving only clean, uncomplicated cases, a strength of the current study is a patient population comprised of all centers for disease control and prevention (CDC) wound classes, including some complex and comorbid patients.

## Methods

### Study design

This study was a prospective, multicenter, single-arm clinical study designed to collect data on the performance of an antimicrobial-coated, noncrosslinked, AC-PDM (XenMatrix™ AB Surgical Graft, C. R. Bard / Davol, Inc.) in a cohort of patients up to 24 months post-implantation (ClinicalTrials.gov # NCT02691962 https://clinicaltrials.gov/ct2/show/NCT02691962). The PDM surface is coated with the antibacterial agents Rifampin and Minocycline in a bioresorbable L-Tyrosine succinate polymer carrier^10^, which has been shown to reduce or inhibit microbial colonization of the underlying PDM^13^,^14^. The current study was designed to treat ~75 patients at 12 sites throughout the United States. The study protocol was approved by the Institutional Review Board (IRB) at each institution prior to enrolling patients, and all patients provided informed consent prior to enrolling in the study. Follow-up visits were conducted at 1, 3, 6, 12, 18, and 24 months following surgery. Patients, investigators, and surgeons were not blinded to the study treatment.

### Inclusion/exclusion criteria

Patients 18 years of age or older, diagnosed with a primary or recurrent ventral or incisional midline hernia requiring surgical repair, were screened for eligibility in the study. Study eligibility included the criteria described above, as well as willingness and ability to provide informed consent, ability to place mesh in the retromuscular or intraperitoneal plane, and willingness and ability to undergo open hernia repair and other study procedures as outlined in the study protocol. Patients were excluded from the study if they met any of the following conditions: use of surgical graft as a bridged repair; more than four prior hernia recurrences; contraindication(s) for placement of surgical graft (as defined by the individual operating surgeons); incomplete removal of pre-existing mesh from a prior hernia repair; requirement for more than a single piece of mesh; intact permanent mesh adjacent to the current hernia to be repaired; peritonitis at the time of surgery; active smoking within 2 weeks prior to surgery; clinically significant chronic obstructive pulmonary disease or heart failure (defined as marked limitation in ability or inability to perform activities of daily living); chemotherapy within 12 months prior to surgery or suspected to be placed on chemotherapy during any part of the study; chronic steroid use (>6 mo) or immunosuppressive drugs; BMI greater than 45 kg/m^2^; cirrhosis or ascites; diagnosed collagen disorder; known infection with HIV; clinically significant kidney disease or on haemodialysis or peritoneal dialysis; American Society of Anesthesiology (ASA) Class 4 or 5; life expectancy of less than 2 years at time of enrolment; pregnant, breastfeeding, or planning on becoming pregnant during the course of the study; known sensitivity to porcine products; allergy, history of allergy, or hypersensitivity to tetracyclines or rifamycins; and/or any condition that would preclude the use of the study device or preclude the subject from completing the follow-up requirements of the study.

### Surgical technique

Prophylactic antibiotics were administered according to participating hospital protocol, and patients underwent open ventral hernia repair. If necessary, adhesiolysis was performed, any prior scar resected, and the edges of the rectus fascia resected/debrided until healthy, well-vascularized tissue was identified to ensure proper apposition of the midline. If a retromuscular approach was conducted, the peritoneum or posterior rectus sheath was carefully dissected to create a retromuscular pocket for the AC-PDM. Once the dissection was complete, the peritoneum/posterior rectus sheath was closed, and the graft was placed in the retromuscular space according to the Instructions for Use^10^. If an intraperitoneal approach was conducted, the device was placed intraperitoneally, after proper sizing and positioning to ensure a minimum 5 cm overlap beyond the defect margins. The device was fixated with transfascial sutures^15^, the fascial and subcutaneous layers closed in layered fashion, and finally, the skin was closed with permanent or long-acting resorbable sutures, with 1–2 cm between sutures. Postoperative care, prescription, and nonprescription medication were given according to hospital protocol in a pragmatic system.

### Data collection

Patient follow-up assessments were completed at 1, 3, 6, 12, 18, and 24 months post-implantation. At each study visit, the following procedures were conducted: physical examination to assess surgical site occurrence (SSO), hernia recurrence, or any other complication, as well as the patient’s weight and skin appearance at the hernia repair site. If infection was suspected, a routine culture was obtained and classified according to the CDC guidelines for superficial and deep surgical site infections^16^. Time to return to work and quality of life assessments were administered [i.e. Carolinas Comfort Scale (CCS), Short Form Health Survey (SF-12v2)], and adverse events were documented. A recurrent ventral or incisional midline hernia was defined as any hernia identified or confirmed during any study follow-up visit within ~5 cm of the index hernia. Adverse events were defined as any undesirable clinical event occurring in the abdominal space, as well as any other undesirable clinical events judged to be related to the study device or surgical procedure.

### Study endpoints

The primary endpoint of the study was to quantify SSO in the first 45 days post-implantation, including surgical site infection (SSI), seroma, wound dehiscence, skin necrosis, and fistulas requiring intervention. Secondary endpoints included length of stay, return to work, SSO greater than 45 days post-implantation, rate of hernia recurrence, rate of reoperation due to the index hernia repair, and quality of life assessments (i.e. Carolinas Comfort Scale and SF-12v2) at 1, 3, 6, 12, 18, and 24 months post-implantation.

### Analysis population

The analysis population included all patients who underwent placement of an AC-PDM. Demographics and baseline characteristics were recorded for this population. Data from all investigational sites were pooled for analysis and tested for potential differences in the primary endpoint. A time-to-event analysis (Kaplan–Meier, K–M) was performed for SSO and hernia recurrence. Those lost to follow-up were censored at their last available follow-up. This work complies with the Strengthening the Reporting of Cohort Studies in Surgery guidelines (STROCSS)^17^.

## Results

### Preoperative variables and subject demographics

**Figure 1:**
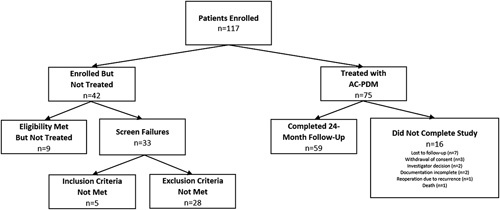
Flow of patients throughout the study period.

As shown in Fig. 1, *n*=117 patients were enrolled in the trial. Of those, a total of *n*=75 patients were treated with AC-PDM, and *n*=59 patients (78.7%) completed follow-up through 24 months. Subject demographics and diagnosis information are shown in Table 1. The majority of the patients were female (*n*=39/75, 52%). The mean age of study patients was 58.6±12.7 years, and the mean BMI was 31.3±4.9 kg/m^2^. Incisional hernias comprised the majority of the cases, with *n*=22 (29.3%) primary incisional hernias and *n*=26 (34.7%) recurrent incisional hernias. The majority of patients (*n*=51, 68%) presented with hernias in multiple locations, which were repaired simultaneously with a single piece of AC-PDM, while a smaller number presented with a hernia in a single location (*n*=24, 32%). Hernia locations ranged from umbilical (*n*=51) to epigastric (*n*=47), infraumbilical (*n*=43), subxiphoid (*n*=23), and suprapubic (*n*=18), and the majority were located in the same area as a prior incision (86.7%). The average hernia defect length was 16.4±8.0 cm, and the average hernia defect width was 10.4±5.3 cm (Table 2). When multiple defects were repaired, the hernia defect dimensions were calculated as the sum of all lengths or the sum of all widths, respectively. The majority of patients presented with comorbidities, mostly commonly, obesity (61.3%), cigarette smoking (48.0%), and diabetes (24.0%).

**Table 1:** Preoperative data: patient demographics and hernia data

Patients treated with AC-PDM, n	75
Patients with 24-month follow-up, *n* (%)	59 (78.7)
Sex	
Male, *n* (%)	36 (48.0)
Female, *n* (%)	39 (52.0)
Race	
Asian, *n* (%)	1 (1.3)
Black, *n* (%)	11 (14.7)
White, *n* (%)	60 (80.0)
Other—Hispanic, *n* (%)	2 (2.7)
Other—Mexican–American, *n* (%)	1 (1.3)
Age (years), mean±SD	58.6±12.7
Body mass index, kg/m^2^ (mean±SD)	31.3±4.9
Comorbidities	
Obesity, *n* (%)	46 (61.3)
Cigarette smoking, *n* (%)	36 (48.0)
Diabetes, *n* (%)	18 (24.0)
Coronary artery disease, *n* (%)	7 (9.3)
Advanced age, *n* (%)	5 (6.7)
COPD, *n* (%)	4 (5.3)
Renal insufficiency, *n* (%)	3 (4.0)
Chronic corticosteroid use, *n* (%)	1 (1.3)
Hernia diagnosis	
Primary ventral, *n* (%)	14 (18.7)
Primary incisional, *n* (%)	22 (29.3)
Recurrent ventral, *n* (%)	13 (17.3)
Recurrent incisional, *n* (%)	26 (34.7)
Hernia location	
Umbilical, *n*	51
Epigastric, *n*	47
Infraumbilical, *n*	43
Subxiphoid, *n*	23
Suprapubic, *n*	18

AC-PDM, acellular porcine dermal matrix; COPD, chronic obstructive pulmonary disease.

**Table 2:** Perioperative data: mesh / defect sizes and surgical technique

Surgical technique	
Retrorectus with CST, *n* (%)	28 (37.3)
Retrorectus without CST, *n* (%)	19 (25.3)
Intraperitoneal with CST, *n* (%)	12 (16.0)
Intraperitoneal without CST, *n* (%)	16 (21.3)
Defect	
Length, cm (mean±SD)	16.4±8.0
Width, cm (mean±SD)	10.4±5.3
Swiss Cheese defect, *n* (%)	21 (28.0)
Mesh	
Length, cm (mean±SD)	22.0±7.1
Width, cm (mean±SD)	17.3±5.6
Fixation	
Long-term absorbable monofilament, *n* (%)	69 (92.0)
Permanent monofilament, *n* (%)	1 (1.3)
Long-term absorbable multifilament, *n* (%)	2 (2.7)
Permanent multifilament, *n* (%)	3 (4.0)
Midline closure	
Sutures, *n* (%)	74 (98.7)
Staples and Sutures, *n* (%)	1 (1.3)
Midline closure spacing of fixation, cm (mean±SD)	0.9±0.4
Midline suture type	
Permanent, *n* (%)	2 (2.7)
Long-term absorbable, *n* (%)	51 (68.0)
Combination, *n* (%)	2 (2.7)
Midline fascia closed completely, *n* (%)	74 (98.7)
Drains	
0 drains, *n* (%)	4 (5.3)
1 drain, *n* (%)	16 (21.3)
≥2 drains, *n* (%)	55 (73.4)
Reason for drain	
Index procedure, *n* (%)	67 (89.3)
Reoperation, *n* (%)	14 (18.7)
Average drain duration, days (mean±SD)	18.8±16.1
Longest drain duration, days (mean±SD)	21.2±17.0
Negative pressure wound therapy, *n* (%)	8 (10.7)
Closed wound, *n*	3
Open wound, *n*	5
Postoperative CDC wound classification	
Class I (clean), *n* (%)	34 (45.3)
Class II (clean-contaminated), *n* (%)	18 (24.0)
Class III (contaminated), *n* (%)	16 (21.3)
Class IV (dirty-infected), *n* (%)	7 (9.3)

CDC, centers for disease control and prevention; CST, component separation technique.

### Operative characteristics and postoperative data

**Figure 2:**
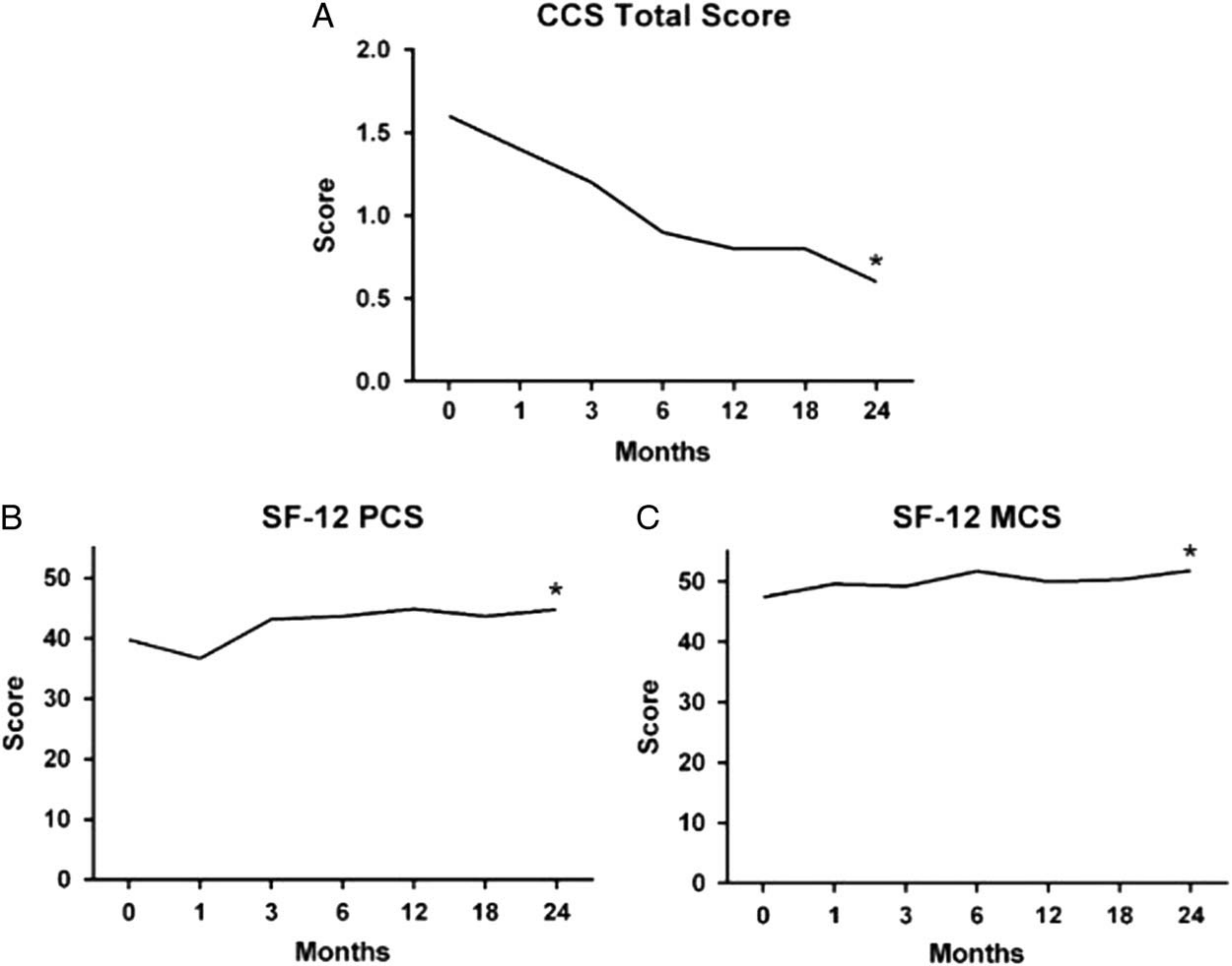
Quality of life assessments: (A) Carolinas Comfort Scale (CCS)—total score (mean);. (B) SF-12v2 Physical Component Score (PCS) (mean); (C) SF-12v2 Mental Component Score (MCS) (mean); *P<0.05 (Baseline vs. 24 months).

The surgical techniques used to place the AC-PDM are detailed in Table 2. A total of *n*=47 (62.7%) AC-PDM grafts were placed in the retromuscular position (*n*=28, 37.3% with component separation technique (CST) and *n*=19, 25.3% without CST). A total of *n*=28 (37.3%) AC-PDM grafts were placed in the intraperitoneal position (*n*=12, 16.0% with CST and *n*=16, 21.3% without CST). Cases requiring CST were split evenly between anterior (*n*=21) and posterior (*n*=19) techniques. Swiss cheese defects (28.0%) and fistula present at the time of surgery (20%) were observed among study patients. Drains were placed in the majority of patients (*n*=71, 94.7%) with a mean duration of 18.8±16.1 days. Postoperative CDC wound class designations are shown in Table 2. There were *n*=34 (45.3%) study patients with a Class I CDC wound class designation, *n*=18 (24.0%) with Class II, *n*=16 (21.3%) with Class III, and *n*=7 (9.3%) with Class IV. Primary and secondary endpoints are reported in Table 3, including hernia recurrence (K–M: 5.8%), definitely device-related adverse events (4.0%), and reoperation (10.7%). There was only one case of mesh infection (*n*=1; 1.3%) which occurred in the first 30 days post-implantation, and none of the AC-PDM grafts were removed at reoperation (*n*=0; 0.0%). A Kaplan–Meier analysis showed 14.7% (*n*=11) SSO in the first 45 days post-implantation, which remained consistent through 24 months post-implantation (*n*=20; 20.0%). All quality-of-life metrics showed significant improvement at 24 months post-implantation (Fig. 2 and Table 3) compared with baseline values (*P*<0.05 in all cases). Hernia-related surgical complications were evaluated according to the Clavien–Dindo system^18^. There were very few grade IVa, IVb, and V hernia-related complications (*n*=3/105; 3%). Just over one third of the hernia-related complications were judged to be grades IIIa or IIIb (*n*=39/105; 37%). The most common hernia-related complications were grade I (*n*=63/105; 60%), with the majority attributed to seroma (*n*=14), bowel obstruction (*n*=9), pain (*n*=8), Ileus (n=4), incisional cellulitis (*n*=4), and SSI (*n*=4).

**Table 3:** Primary and secondary study endpoints

SSO	n	K-M
SSO ≤45 day	11	14.7% (95% CI =8.4%, 24.9%)
SSO >45 day	20	20.0% (95% CI =11.6%, 30.8%)
Recurrence:	*n*	K–M
Hernia recurrence rate	4	5.8% (95% CI =2.2%, 14.8%)
Other endpoints:		
Surgical procedure time, min (mean ± SD)	260.6±115.8	
Length of stay, days (mean±SD)	6.7±6.1	
Return to work, days (mean±SD)	40.8±32.2	
Reoperation rate, *n* (%)	8 (10.7)	
Reason for reoperation		
SSI, *n* (%)	4 (5.3)	
Seroma, *n* (%)	2 (2.7)	
Hernia recurrence, *n* (%)	1 (1.3)	
Wound dehiscence, *n* (%)	1 (1.3)	
Definitely device-related adverse events, *n* (%)	3 (4.0)	
Seroma, *n*	1	
Hernia recurrence*, n*	1	
Abdominal abscess*, n*	1	
	Baseline	24 months
Carolinas Comfort Scale – Total Score (mean±SD)	1.6±1.65	0.6±1.01*
SF-12 Physical Component Score (mean±SD)	39.8±10.50	44.8±10.06*
SF-12 Mental Component Score (mean±SD)	47.4±12.31	51.8±10.30*

SSI, surgical site infection; SSO, surgical site occurrence.

*P<0.05 compared to baseline.

## Discussion

The objective of this prospective, multicenter, single-arm study was to evaluate the clinical performance of an AC-PDM graft 45 days and 24 months after implantation in a retromuscular or intraperitoneal location. Patients with ventral or incisional midline hernias were included, along with all CDC wound classes. The results of this study showed a relatively low rate of SSO requiring intervention within the first 45 days post-implantation (K–M: 14.7%), including SSI, seroma, wound dehiscence, skin necrosis, and fistula. Throughout the long-term, 24-month follow-up period, SSO requiring intervention (K–M: 20.0%) and reoperation (10.7%) were comparable to other published studies^19^–^22^. Recurrence (K–M: 5.8%) and definitely device-related adverse events (4.0%) remained low, while quality of life metrics demonstrated significant improvement over baseline.

The results of the current study compare well with several published studies that have evaluated clinical outcomes associated with the previously-developed, uncoated version of PDM (XenMatrix™). This earlier version lacks the antimicrobial coating found on the newer AC-PDM study device (XenMatrix™ AB)^19^–^21^. In 2010, Pomahac and colleagues published the results of a study in which PDM was implanted in an underlay fashion in patients distributed across all wound classes. They reported 7% hernia recurrence, 7% SSI, and 21% seroma associated with uncoated PDM at a mean follow-up of 16.5 months^19^. When their longer-term follow-up of 40 months was published in 2013, similar results were again reported (7.9% hernia recurrence, 5.2% SSI, and 21% seroma)^20^. Another group, Byrnes and colleagues, implanted XenMatrix™ as an underlay mesh with fascial closure in 84% of cases and CST in 36% of cases. They reported 7.2% hernia recurrence, 0% SSI, and 0% seroma at a mean follow-up of 30.6 months^21^. The results of these retrospective studies compare well with the current multicenter prospective study in which the AC-PDM exhibited 5.8% (K–M) recurrence and 20.0% (K–M) SSO at 24 months. The slightly higher rate of recurrences in the uncoated PDM studies is likely related to the longer follow-up period associated with those studies (30.6 and 40.1 months) compared with the 24-month follow-up of the current study. All of these studies were performed in a similar patient population comprised of a mixture of CDC wound classes, but predominantly Class I, clean cases.

Although biological tissue-derived materials are not currently indicated for use in contaminated or infected fields, these materials have occasionally been used in an “off label” fashion at the discretion of the operating surgeon. Two such studies reported the clinical results associated with the same AC-PDM utilized in the current study, but implanted in contaminated or infected fields^23^,^24^. In a third study, another type of noncrosslinked, uncoated PDM (Strattice™, LifeCell Corporation) was investigated in a clinical trial (RICH Trial) involving patients with contaminated or infected wounds^22^. The results of these off-label studies show generally higher hernia recurrence rates relative to the current study, highlighting the potential benefits of an antimicrobial coating if an implanted material encounters contamination. The antimicrobial coating may protect the underlying scaffold from rapid degradation and loss of mechanical strength, both of which could lead to higher recurrence rates in uncoated devices exposed to the same wound environment. To extend these conclusions, a comparative study with similar percentage of contaminated cases should be undertaken.

The strengths of the current study include the prospective, multicenter design and the mixture of wound classes represented in the complex, comorbid patient population. This study population is representative of a typical patient population encountered by surgeons. However, the current study is not without limitations. Notably, this is a single-arm study without a control group, making it impossible to directly compare the results of this AC-PDM with another biomaterial. Additionally, the patient population was comprised of predominantly CDC Class I wounds, making it difficult to assess how AC-PDM may perform in predominantly contaminated or infected wound environments.

Future studies should include a randomized controlled study design with a defined control group in order to directly compare the results of AC-PDM to another implantable material. Additionally, while *n*=75 patients is a moderately large cohort, an even larger number of study patients would allow for further statistical sub-analyses, with the potential to yield additional clinical insights beyond the current study results. Finally, a longer, 3–5 year follow-up period could provide further insight into recurrence rates and other outcomes at time periods well beyond the complete remodelling of the PDM, when it is no longer contributing mechanical strength to the repair site.

## Conclusion

In conclusion, this prospective, multicenter, single-arm study demonstrated favourable results for an antimicrobial-coated, noncrosslinked porcine acellular dermal matrix graft in patients with ventral or incisional midline hernias, across all CDC wound classes. Low incidence of hernia recurrence (5.8%) and definitely device-related adverse events (4.0%) were observed with reoperation (10.7%) and SSO (K–M: 14.7% ≤45 days and 20.0% >45 days) comparable to other published studies.

## Ethical approval

The study protocol was approved by the Institutional Review Board (IRB) at each institution prior to enrolling patients, and all patients provided informed consent prior to enroling in the study. (ClinicalTrials.gov # NCT02691962 https://clinicaltrials.gov/ct2/show/NCT02691962 )

## Consent

The study protocol was approved by the Institutional Review Board (IRB) at each institution prior to enrolling patients, and all patients provided informed consent prior to enroling in the study.

## Source of funding

This project was sponsored by Davol Inc. (Warwick, RI), a subsidiary of C. R. Bard, Inc. (Franklin Lakes, NJ). Bard and Davol have joined BD (Franklin Lakes, NJ).

## Conflicts of interest disclosure

All conflicts of interest have been declared in the Author Disclosure Form uploaded with this submission and are listed below in a de-identified list without author names to preserve anonymity.

Ilahi’s institution received a grant from C. R. Bard, Inc./Davol/Becton Dickinson (BD) to support the work under consideration. Ilahi has no other financial conflicts of interests to disclose relevant to the current study or any other outside work.

Velmahos has other financial conflicts of interests to disclose relevant to the current study or any other outside work.

Janis’s institution received a grant from C. R. Bard, Inc./Davol/Becton Dickinson (BD) to support the work under consideration. Janis also reports publishing royalties from Thieme and Springer and consulting fees from Allergan/LifeCell.

Kovach has no financial conflicts of interests to disclose relevant to the current study. Kovach has received payment for lectures from C. R. Bard, Inc./Davol/Becton Dickinson (BD), W.L. Gore & Associates, and Integra outside the current work.

McLean has no financial conflicts of interests to disclose relevant to the current study or any other outside work.

Askari’s institution received a grant from C. R. Bard, Inc./Davol/Becton Dickinson (BD) to support the work under consideration. Askari has no other financial conflicts of interests to disclose relevant to any other outside work.

Sommer has no financial conflicts of interests to disclose relevant to the current study or any other outside work.

Dr. Agarwal’s institution received a grant from C. R. Bard, Inc./Davol/Becton Dickinson (BD) to support the work under consideration. Dr. Agarwal has no other financial conflicts of interests to disclose relevant to the current study or any other outside work.

Srinivasan has no financial conflicts of interests to disclose relevant to the current study or any other outside work.

Wong has no financial conflicts of interests to disclose relevant to the current study. Wong has received consulting fees (A Cell, Inc.), stock ownership (Lymphagen Corporation), and grants (A Cell, Inc.) outside the current work.

Pakula has no financial conflicts of interests to disclose relevant to the current study. Pakula reports consulting fees and speaking fees from Becton Dickinson, Intuitive Surgical, and Medtronic for work outside the current study.

Miranda’s institution received a grant from C. R. Bard, Inc./Davol/Becton Dickinson (BD) to support the work under consideration. Miranda also reports consulting fees (RTI Surgical, ReNerve, and Allergan, Abominal Wall Advisory Board) equity interest (Additive Orthopaedics),

honoraria/speaking fees (Integra Life Sciences, Candela Corporation, and MiMedx), and patient enrolment bounties (C. R. Bard, Inc./Davol/Becton Dickinson (BD) and Sientra) outside the current work.

Stringer has no financial conflicts of interest to disclose relevant to the current study or other outside work.

Breen’s institution received a grant from C. R. Bard, Inc./Davol/Becton Dickinson (BD) to support the work under consideration. Breen has no other financial conflicts of interests to disclose relevant to the current study or any other outside work.

Shapiro has no financial conflicts of interests to disclose relevant to the current study or any other outside work.

McGraw has no financial conflicts of interests to disclose relevant to the current study or any other outside work.

Bascone has no financial conflicts of interests to disclose relevant to the current study or any other outside work.

Deeken received consulting fees to support manuscript generation for the work under consideration from Becton Dickinson. Deeken reports consulting fees (Becton Dickinson, Ethicon, Medtronic, Osteogenics, Polynovo, Surgical Innovation and Associates, Surgimatrix, TelaBio, and Tissum) for work outside the current study, and ownership/employment (Covalent Bio LLC). Deeken also holds the following issued patents related to hernia-repair materials: 2009293001, 2334257, 2334257UK, 602009046407.8, 2334257FR, 16/043849, 2737542.

Martindale’s institution received a grant from C. R. Bard, Inc./Davol/Becton Dickinson (BD) to support the work under consideration. Martindale has no personal financial conflicts of interests to disclose relevant to the current study. Martindale reports consulting fees (Allergan, and Nestle), employment (Oregon Health & Science University), grants, and speakers’ fees (Allergan, Nestle, Fresenius, and Abbott) for work outside the current study.

## Research registration unique identifying number (UIN)

1. Name of the registry: ClinicalTrials.gov
2. Unique Identifying number or registration ID: # NCT02691962
3. Hyperlink to your specific registration (must be publicly accessible and will be checked): https://clinicaltrials.gov/ct2/show/NCT02691962

## Provenance and peer review

Not commissioned, externally peer-reviewed.
